# Anti-Adhesive Surfaces Inspired by Bee Mandible Surfaces

**DOI:** 10.3390/biomimetics8080579

**Published:** 2023-12-01

**Authors:** Leonie Saccardi, Jonas Schiebl, Franz Balluff, Ulrich Christ, Stanislav N. Gorb, Alexander Kovalev, Oliver Schwarz

**Affiliations:** 1 Institute of Biomaterials and Biomolecular Systems, University of Stuttgart, 70569 Stuttgart, Germany; 2 Department of Biomechatronic Systems, FraunhoferInstitute for Manufacturing Engineering and Automation IPA, 70569 Stuttgart, Germany; 3 Department of Applied Coating Technology, Fraunhofer-Institute for Manufacturing Engineering and Automation (IPA), 70569 Stuttgart, Germany; 4 Department Functional Morphology and Biomechanics, Zoological Institute, Kiel University, 24118 Kiel, Germany

**Keywords:** adhesion, propolis, biomimetics, mandibles, honeybee

## Abstract

Propolis, a naturally sticky substance used by bees to secure their hives and protect the colony from pathogens, presents a fascinating challenge. Despite its adhesive nature, honeybees adeptly handle propolis with their mandibles. Previous research has shown a combination of an anti-adhesive fluid layer and scale-like microstructures on the inner surface of bee mandibles. Our aim was to deepen our understanding of how surface energy and microstructure influence the reduction in adhesion for challenging substances like propolis. To achieve this, we devised surfaces inspired by the intricate microstructure of bee mandibles, employing diverse techniques including roughening steel surfaces, creating lacquer structures using Bénard cells, and moulding resin surfaces with hexagonal patterns. These approaches generated patterns that mimicked the bee mandible structure to varying degrees. Subsequently, we assessed the adhesion of propolis on these bioinspired structured substrates. Our findings revealed that on rough steel and resin surfaces structured with hexagonal dimples, propolis adhesion was significantly reduced by over 40% compared to unstructured control surfaces. However, in the case of the lacquer surface patterned with Bénard cells, we did not observe a significant reduction in adhesion.

## 1. Introduction

In many industries, unwanted adhesion presents a significant challenge, often leading to costly and environmentally harmful cleaning procedures. One notable example of unwanted adhesion in technology pertains to the accumulation of resins on the cutting edges of woodworking tools. This issue served as the catalyst for a top-down bionic development process [1,2]. Leveraging inspiration from nature’s anti-adhesive strategies can yield innovative solutions to technical challenges [3,4,5,6,7].

In the biomimetic development process, the initial step involves abstracting a specific problem to identify analogous challenges in the natural world [8]. The ability to prevent adhesion to surfaces or safeguard the functionality of organs from adhesion has been a selective factor in biological evolution, significantly influencing survival probabilities.

Therefore, one search strategy entails identifying adhesive substances—whether self-produced or acquired from the environment—with which certain animal surfaces regularly come into contact.

Propolis, a resin-based substance with adhesive properties, is used by honeybees to seal cracks and smooth internal hive walls [9,10]. Remarkably, honeybees appear to have no adverse reactions when in contact with propolis. They adeptly manipulate finished propolis and gather key resin components from plant buds without any issues. Propolis exhibits strong adhesion to various substrates, including glass, steel, and even PTFE, as demonstrated by our previous study [11]. Notably, in wet conditions, propolis adhesion is significantly reduced. Furthermore, our earlier findings indicate a positive correlation between temperature and propolis adhesion.

There is vast literature on Hymenoptera-mandibles [12,13,14]. However, a detailed anatomical investigation of honeybee mandibles, which frequently come into contact with sticky propolis and plant resins, was only recently conducted [15].

Of particular interest is the medial surface of the mandible, which plays a crucial role in propolis processing, given its frequent contact with the sticky material during biting and shaping [16,17].

**Figure 1:**
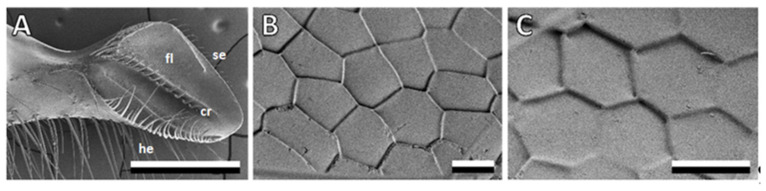
Bee mandibles washed with chloroform and water. (**A**–**C**): SEM micrographs of bee mandibles (mandible overview and close-up of flat area). cr, central ridge; fl, flat area; he, hairy edge; se, sharp edge. Scale bars: 500 μm (**A**), 10 μm (**B**,**C**).

Bee mandibles have a sturdy stem at the base and a concave, spoon-shaped tip at the other end, which is crucial for processing propolis (Figure 1A) [18,19]. The spoon-shaped tip tapers towards the apex and has a hairy edge on one side, with long, flexible hairs covering over half of the medial surface, while the opposite side has a sharp, hairless edge. A central ridge runs along the medial surface, fading towards the apex, adorned with stiff, grooved bristles curving toward the sharp edge. There is an elevated ledge adjacent to the hairy edge, forming a channel between the central ridge and the ledge, extending from the stem to the apex. On the other side of the central ridge, a flat area extends to the sharp edge [15].

It was observed that propolis adhesion is significantly lower on bee mandibles in comparison to other surfaces, such as glass [15]. This observation suggests that bees may have evolved an anti-adhesive strategy to facilitate propolis handling, preventing contamination with resin.

There are several hypotheses explaining the mechanisms underlying the reduction in adhesion based on principles observed in other insects. Voigt et al. [20] suggest the following potential strategies to reduce adhesion on insect cuticles: (1) specific surface chemistry; (2) surface microstructures: for example, water striders utilise complex two-level microstructures, including microtrichia, to create a super-hydrophobic surface that reduces adhesion [21] and (3) the presence of an easy-to-break solid or fluid layer preventing strong bonding through cohesion failure, like the wax layers on *Nepenthes alata* pitchers that prevent insect adhesion by breaking off [22].

Bornean stingless bees (Hymenoptera, Meliponini), like honeybees, collect resin for nest construction and protection against intruders [23]. They can remove resin from their mandibles without any residue, leading to the suggestion that these bees may use temporary lubrication to reduce resin adhesion on their mandibles [24,25].

Our recent findings indicate that a combination of surface structure and a fluid layer plays a significant role in reducing propolis adhesion on bee mandibles [15]. The removal of the liquid layer from the cuticle surface using substances like chloroform or acetone resulted in a substantial increase in propolis adhesion [15]. Consequently, we have hypothesised that this fluid serves to lubricate the mandible and minimise propolis adhesion.

The medial surface of bee mandibles also features hexagonal or pentagonal microstructures with dimensions ranging from 10–20 µm [15]. Our findings suggest that these structures alone do not independently reduce propolis adhesion; their anti-adhesive effect is achieved only in combination with fluid [15]. The functional significance of these microstructures likely lies in maintaining a specific thickness of the fluid layer and facilitating the spreading of the fluid across the surface. It is worth noting that the microstructures exhibit a slight asymmetry in shape depending on their location on the mandible, and the step height between neighbouring microscales measures 0.7 µm.

To better understand and replicate the anti-adhesive strategy of bee mandibles, we conducted a series of experiments on various substrates. These substrates were engineered to mimic the patterns found on bee mandibles, and their anti-adhesive properties were assessed through adhesion experiments. This approach aimed to gain deeper insights into the influence of material surface chemistry and structure on propolis adhesion.

## 2. Materials and Methods

### 2.1. Propolis

Raw propolis was provided by one of the co-authors (O.S., Stuttgart, Germany). The homogenisation process followed the procedure outlined in [3]. Propolis chunks were homogenised by mixing, finely ground, and subsequently stored at −20 °C. The pulverisation method employed here is akin to that commonly used for producing propolis extract [26]. To prevent contamination, all handling of the propolis was carried out using gloves that had been thoroughly cleaned with ethanol (Rotipuran^®^, ≥99.8%, p.a., Carl Roth GmbH & Co. KG, Karlsruhe, Germany).

### 2.2. Imaging of Bee Mandibles

The mandibles of honeybees (*Apis mellifera*) were prepared and examined following the procedure outlined in Saccardi et al. [15]. They were initially cleaned with a solution of chloroform and water and then examined using a scanning electron microscope (SEM) (Hitachi S-4800, Hitachi High-Technologies Corp., Tokyo, Japan) with a 3 kV accelerating voltage. Fractures of frozen bee mandibles were further analysed using cryo SEM. Additionally, a confocal 3D laser scanning microscope was used to measure the profiles of the surface structures on the mandibles.

### 2.3. Development and Characterisation of Bioinspired Substrates

To examine the influence of surface structures on propolis adhesion across various materials, structured substrates from steel, Spurr’s resin, and UV lacquer were prepared using diverse methods for subsequent adhesion experiments. Unstructured substrates composed of the same materials served as our control group.

Rough steel: A section of an unpolished steel circular saw blade (1.2003, Fraunhofer IPA, Stuttgart, Germany) was used as a rough substrate. As a contrasting smooth control, a steel plate (1.4016, Abrams Premium Stahl^®^, Osnabrück, Germany) was polished using a polishing machine (Minitech 233, Presi, Eybens, France). The polishing involved the successive use of aluminium oxide suspensions with descending particle sizes (12, 3, 1, and 0.3 µm).

Bénard structures: Rayleigh–Bénard convection is a phenomenon in fluid dynamics that occurs when a layer of fluid is heated from below and cooled from above, resulting in the formation of characteristic convection cells [27,28]. These convection flows encompass Bénard cells, driven by density differences, and Marangoni cells, driven by surface tension disparities. The two effects occur in combination, with one of the two effects usually predominating. The resulting cells can form mountains and valleys, which are “frozen” by radiation curing [29]. We used this phenomenon to create structured surfaces by applying an UV lacquer (Cymel 328 with the addition of 23% isopropanol) to a metal plate. This lacquer-coated plate was structured using Rayleigh–Bénard convection, generated by heating it from the uncoated side. Once the cells reached the desired size, the lacquer was solidified using UV light (M-25-2*1-TR-SS, IST Metz, Nürtingen, Germany). For the smooth control substrate, the lacquer was applied without structuring and polymerised following application.

Hexagons: Hexagonal microstructures resembling those found on tarsal attachment pads of the bush cricket *Tettigonia viridissima* were initially described and replicated by Varenberg and Gorb [30,31]. These structures, 10 µm in diameter, closely resemble those present on bee mandibles. Negative and positive steel templates (OVD Kinegram, Zug, Switzerland) of these hexagonal structures with a 10 µm diameter were used to produce replicas of the pattern employing the two-step moulding method described by Gorb [32] and Koch et al. [33].

To ensure the success of the moulding process, some of the resulting substrates made from Spurr’s epoxy resin (Spurr’s low viscosity kit, Plano, Wetzlar, Germany) were sputter-coated with a 10 nm thick layer of gold–palladium and subsequently examined using a scanning electron microscope (SEM) (Hitachi S-4800, Hitachi High-Technologies Corp., Tokyo, Japan) at 3 kV accelerating voltage. The surfaces of these Spurr substrates exhibited either hexagon-shaped dimples or hexagon-shaped pins. The dimples had a contact area fraction of 25%, while the hexagonal pins had a contact area fraction of 75% [31]. As a smooth resin control surface, a resin replica of a clean glass surface was prepared following the two-step moulding method described by Gorb [32] and Koch et al. [33].

The surface free energy of all samples was characterised by measuring the contact angles of water on the substrates using the sessile drop method with a 2 µL drop volume. This was done with an optical contact angle measuring system (OCAH200, DataPhysics Instruments GmbH, Filderstadt, Germany). Between 5 and 10 contact angle measurements were conducted for each substrate.

The substrates produced through the three different methods were examined using a confocal 3D laser scanning microscope (Keyence VK-X250; Keyence Corporation, Osaka, Japan). MultiFileAnalyzer software (Version 1.2.6.106, Keyence Corporation, Osaka, Japan) was employed to measure the profiles and dimensions of surface features.

### 2.4. Adhesion Measurements on Structured/Bioinspired Substrates

Adhesion experiments were performed with propolis samples on structured and unstructured substrates. Prior to each adhesion experiment, a small quantity of propolis powder was defrosted and thoroughly blended by kneading to achieve a uniform consistency. Cone-shaped propolis specimens with a spherical tip were then manually crafted. The samples’ surface characteristics were examined with a fast-scanning 3D measurement microscope (Keyence VR 3100; Keyence Corporation, Osaka, Japan). The samples’ profiles were measured at five locations positioned in a star-like pattern extending through the apex. To approximate the radiuses at the tips of the samples, circles were matched to the profiles of the samples in five distinct orientations. The radii of these circles were measured and subsequently averaged. The effective elastic modulus and the pull-off force of propolis were measured with a microforce measurement device (Basalt-BT01; Tetra GmbH, Ilmenau, Germany [34,35,36]).

The setup comprised a fibre-optical sensor and micromanipulators serving as a base for securing the substrate material, along with a metal spring (utilising springs with spring constants of 618 and 539 N/m). The piezo drive was responsible for vertically displacing the sensor along with the spring, applying force during the loading phase and releasing it during the unloading of the sample.

A shortened glass capillary (5 μL micropipette Blaubrand R© Intra END, BRAND GMBH + CO KG, Wertheim, Germany) was attached to the metal spring with cyanoacrylate glue. The cone-shaped propolis sample was affixed to the end of the capillary without any additional adhesive. The test substrates were securely attached to the micromanipulator platform using double-sided adhesive tape. The propolis sample was brought into contact with the substrate, and it was withdrawn from the surface as soon as the applied force reached 5 mN. The load was chosen to approximate the load exerted by bees when handling propolis. Given the absence of specific studies on mandibular forces and pressures of honeybees, we used pressure values previously measured at the tip of the mandibles in predacious Coleoptera [37] as a reference for the load applied to the propolis sample. Our experiments were performed in a quasi-static regime (at a very slow velocity) and that is why the velocity did not play an important role in our results.

Propolis adhesion was tested on rough steel, Spurr with hexagonal pins and hexagonal dimples, UV lacquer structured with Bénard cells, and their respective unstructured control surfaces. For each propolis sample, a set of 11 single measurements was performed, each on a different spot of the substrate (N = 5 propolis samples, *n* = 11 measurements per sample). The maximum loading force was 5 mN. All experiments were carried out at room temperature (24 ± 0.5 °C) with a relative humidity of 37 ± 9%.

Following the 11 measurements on the structured substrate materials, we performed an additional set of five reference measurements on the unstructured control surface made from the same material.

After the adhesion experiments, the substrate material was examined under a binocular microscope (Leica M205 A, Leica Microsystems GmbH, Wetzlar, Germany) to identify any potential propolis residues or prints in the contact area.

### 2.5. Data Analysis and Statistics

Adhesion experiments were evaluated as described bySaccardi et al. [3] using Matlab (version R2015b, The MathWorks, Inc., Natick, MA, USA).

Statistical analysis was carried out using R software, version 3.6.1 (The R Foundation for Statistical Computing). The data were initially tested for normal distribution and variance homogeneity using the Kolmogorov–Smirnov and Levene’s tests, respectively. Subsequently, the comparison of propolis adhesion on different substrates was executed with a one-way ANOVA, complemented by a pairwise multiple comparison procedure (Tukey test).

To explore potential correlations between the Young’s modulus and work of adhesion obtained from the adhesion experiments, the Pearson’s correlation coefficient was calculated.

### 2.6. Image Processing

SEM images were processed using Gimp (version 2.10.14, Spencer Kimball, Peter Mattis and the GIMP Development Team). The adjustments, like fine-tuning colour levels, contrast, and brightness, were uniformly applied to the entire image. Furthermore, scale bars and labels were added.

Profiles acquired through the confocal 3D laser scanning microscope were digitised using WebPlotDigitizer (version 4.2, https://automeris.io/WebPlotDigitizer, accessed on 25 January 2020, Ankit Rohatgi, San Francisco, CA, USA).

## 3. Results

### 3.1. Bee Mandible Surfaces

**Figure 2:**
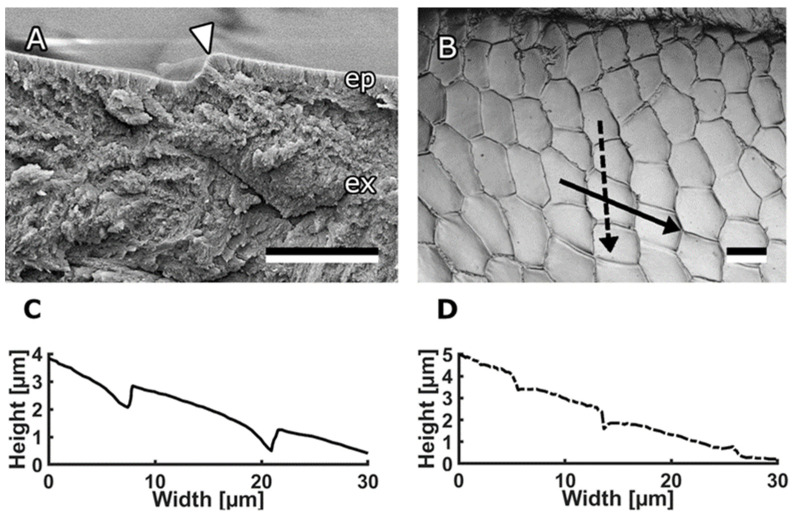
Profile of scales on bee mandibles. (**A**) Cryo-SEM micrograph of mandible cuticle. The arrowhead indicates the step between two scales. (**B**) 3D laser scanning microscope image of medial surface of the mandible within the channel area. (**C**) Profile of the scales along the solid arrow in (**B**). (**D**) Profile of the scales along the dashed arrow in (**B**). Scale bars: 2 μm (**A**), 10 μm (**B**). Published by Beilstein J Nanotechnol., distributed under the terms of the Creative Commons Attribution 4.0 International License, https://creativecommons.org/licenses/by/4.0).

Honeybee mandibles, specifically the structures on the medial surface, served as a source of inspiration for the creation of bioinspired surfaces. These mandibles exhibit a distinct morphology, featuring a robust stem at the proximal end and a concave, spoon-shaped tip at the distal end (Figure 1A). The focal point of interest lies in the medial surface of this tip, which is covered with anisotropic scale-like micropatterns (Figure 1B,C). Most of these scales on the medial surface of the mandible are either pentagonal or hexagonal, although the specific shape and proportions vary depending on their location. In the flat area, extending from the central ridge toward the sharp edge, the scales measure 18.1 ± 2.4 μm in length and 9.55 ± 1.3 μm in width at their minimum and maximum points, respectively. In the channel area, extending from the central ridge toward the hairy edge, the structures exhibit a rounder shape, measuring 9.34 ± 0.77 μm in length and 8.21 ± 0.82 μm in width, with blunter edges. To comprehensively study the topography of these structures, we used cryo-SEM micrographs of fractured mandibles (Figure 2A) and a 3D laser scanning microscope (Figure 2C,D). The scales themselves are either flat or slightly convex, and they either overlap or form steps between them. In most areas of the mandible, the step height between the scales was consistently measured to be 0.71 ± 0.26 μm.

### 3.2. Characterisation of Bioinspired Surfaces

#### 3.2.1. Rough Steel

**Figure 3:**
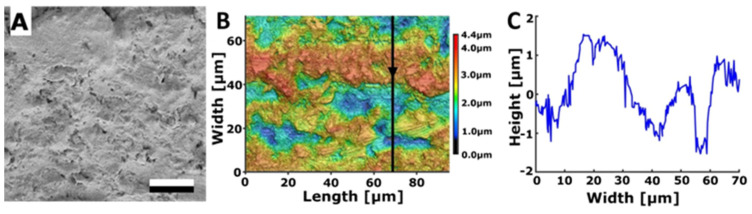
Surface of the rough steel substrate. (**A**) SEM micrograph. Scale bar: 10 μm. (**B**) 3D topography. (**C**) 2D height profile along the arrow in subplot B.

As the initial candidate for a biomimetic surface with anti-adhesive characteristics, a substrate with relatively random surface structures—a sand-blasted steel plate—was chosen. This steel surface displayed an irregular pattern characterised by a series of hills and valleys (Figure 3). The surface roughness of the sand-blasted steel plate was measured to be Sa = 0.708 ± 0.155 µm. Water droplets had a contact angle of 89.7° ± 3.2° on this rough steel surface. In contrast, a polished control surface, also composed of steel, showcased significantly lower roughness, with Sa = 0.038 ± 0.013 µm and a contact angle of 82.6° ± 1.4° for water.

#### 3.2.2. Bénard Structures

**Figure 4:**
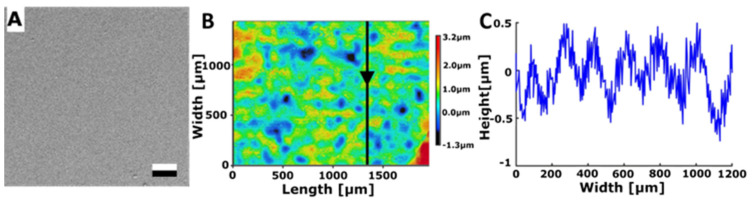
Surface structured with Bénard cells. (**A**) SEM micrograph. Scale bar: 10 μm. (**B**) 3D topography. (**C**) 2D height profile along the arrow in subplot B.

Another approach for creating a surface pattern inspired by the structures found on bee mandibles involved structuring UV lacquer using Rayleigh–Bénard convection with appropriately sized Bénard cells. To achieve this, a metal plate coated with UV lacquer was heated from below, and polymerisation was initiated once the Bénard cells attained a size of approximately 150–200 µm in width and 0.5–1.0 µm in height (Figure 4). This process of Rayleigh–Bénard convection yielded a regular pattern of hills and valleys. The surface roughness of the surface structured with Bénard cells was measured to be Sa = 0.532 ± 0.211 µm. The water contact angle on this structured surface measured 80.6° ± 3.0°.

In contrast, for the control substrate, the UV lacquer was not structured before undergoing polymerisation and the water contact angle on this smooth surface was 77.1° ± 4.5°.

#### 3.2.3. Hexagons

The microstructures observed on the tarsal attachment pads of the bush cricket *Tettigonia viridissima* resemble those found on bee mandibles in terms of their hexagonal shape and dimensions. These structures were previously described and replicated by Varenberg and Gorb [18,19].

**Figure 5:**
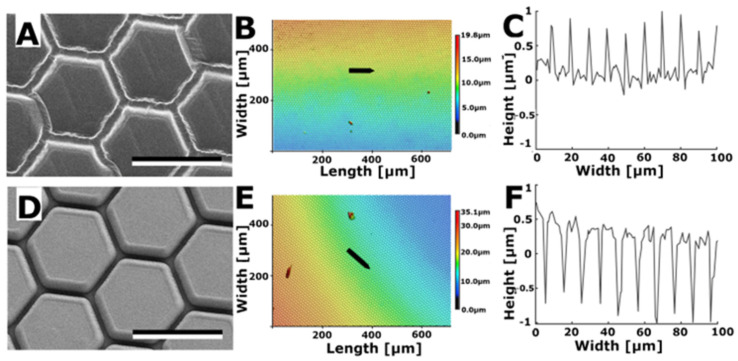
Hexagonal surface structures on a surface made from the Spurr’s resin. (**A**) SEM image of hexagonal dimples. (**B**) 3D topography. (**C**) 2D profile of hexagonal dimples along the arrow in B. (**D**) SEM image of hexagonal pins. (**E**) 3D topography. (**F**) 2D profile of hexagonal pins along the arrow in (**E**). Scale bars: 10 µm (**A**,**D**).

Negative and positive steel templates (OVD Kinegram, Zug, Switzerland) of these hexagonal structures with a 10 µm diameter were used to produce moulds. Subsequently, the moulds were used to create structured substrates with Spurr’s resin. This process resulted in the generation of two variations of hexagonal structures: hexagon-shaped dimples (Figure 5A–C) and hexagon-shaped pins (Figure 5D–F). The hexagons had an approximate diameter of 10 µm. Dimples were set apart by a border ranging from 0.5 to 1.0 µm in height, while pins were surrounded by a trench that reached a depth of 1.0 to 1.5 µm.

The surface roughness of the Spurr surface structured with hexagonal dimples was measured to be Sa = 0.771 ± 0.159 µm and for the surface with hexagonal pins it was Sa = 0.449 ± 0.137 µm. Subsequent measurements revealed that the water contact angle on the Spurr surface with hexagonal dimples was 75.9° ± 5.1°, whereas the contact angle on Spurr with hexagonal pins was 124.1° ± 4.9°. In comparison, the contact angle of water on the smooth control surface composed of Spurr resin was 88.6° ± 3.9°.

### 3.3. Adhesion on Bioinspired Substrates

**Figure 6:**
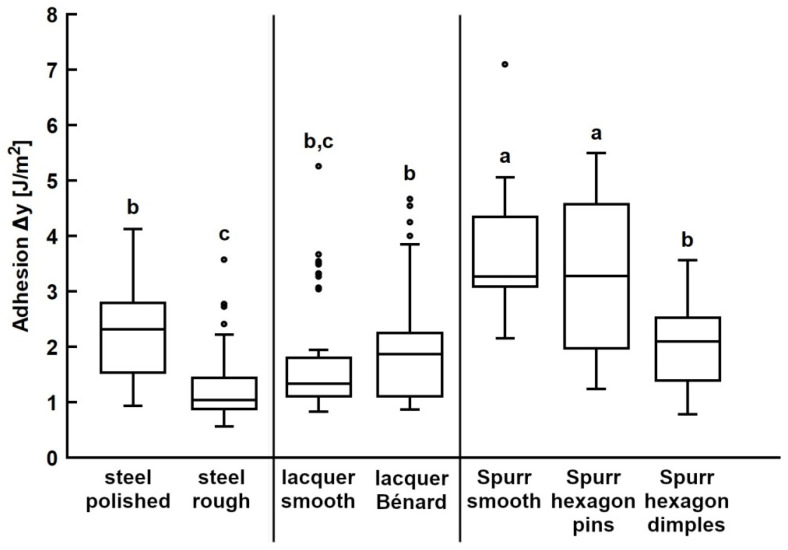
Propolis adhesion on structured substrates. Work of adhesion of propolis samples was tested on different substrate materials structured with bioinspired patterns and on corresponding unstructured control substrates. The ends of the boxes define the 25th and 75th percentiles, with a line at the median and error bars defining the 10th and 90th percentiles. The outlier markings represent individual measurements. Conditions and substrates marked with different letters differ significantly from each other (one-way ANOVA, *p* < 0.001 and Tukey test, *p* < 0.05).

Propolis adhesion was measured on the bioinspired surfaces introduced in this study and their corresponding smooth control surfaces (Table 1). On the rough steel substrate, the measured work of adhesion was 1.26 ± 0.61 J/m^2^ and there was a significant difference between the work of adhesion on the rough and polished steel (Figure 6, left section; *p* < 0.0001).

In the case of adhesion tests conducted with propolis on UV lacquer-coated substrates, there was no significant difference in the work of adhesion between the smooth control and the substrate structured with Bénard cells (Figure 6, middle section; *p* = 0.9997).

Additionally, we assessed propolis adhesion on structured Spurr’s resin substrates and compared it to adhesion on the smooth resin surface (Figure 6, right section). Notably, there was no significant difference in the work of adhesion between the smooth Spurr substrate and the substrate with hexagonal pins (*p* = 0.9920). However, the work of adhesion was significantly lower on substrates structured with hexagonal dimples (*p* < 0.0001).

## 4. Discussion

### 4.1. Development and Characterisation of Bioinspired Surfaces

Various approaches were employed to create structured substrates inspired by the micropatterns observed on bee mandibles. In the initial approach, the bee mandible structures were interpreted as a form of surface roughness, leading to the utilisation of a rough steel surface as a technical approximation. However, the structures on the rough steel surfaces were random in size and distribution, lacking a distinct shape akin to the scales present on the mandible. Consequently, the rough steel surface served as a rather crude approximation of the bee model.

A more regular patterned surface was attained by structuring UV lacquer with Bénard cells. These Bénard cells generated a smooth and regular pattern of valleys and hills. These structures were only visible using a confocal 3D laser scanning microscope rather than a scanning electron microscope (SEM), likely because they were relatively flat and lacked sharp edges. Notably, the lateral dimensions of these structures were approximately ten times larger than those observed on bee mandibles, and they did not possess a distinct geometric shape.

Hexagonal shapes [30,31] proved to be a better match in terms of size and shape when compared to bee patterns. Nevertheless, the hexagonal shapes still differed from the bee patterns as they were isotropic and did not overlap like the scale-like microstructures on bee mandibles. Neither hexagonal pins nor dimples provided a perfect representation of the structures on bee mandibles. However, hexagonal pins appeared to closely resemble the scales on bee mandibles, as these scales often had a relatively flat surface area. Even in instances where some scales in the mandible’s channel area exhibited blunter edges, the overall profile indicated that the bulges did not significantly rise when compared to the profiles of hexagonal dimples, where clear spikes were visible between each valley.

A more promising approach for future replication of the bee mandible pattern might involve the production of a laser-engraved metal template [38,39,40,41,42,43,44,45] based on the 3D data obtained from the mandible surface. This template could then be employed to replicate the structure using a two-step moulding technique.

Additionally, it is worth noting that the chemical composition of materials differed: steel (mostly iron alloy) and epoxy resin (organic compounds) were used. Despite this distinction, both materials exhibited similar surface energies, with dispersive interactions predominating over polar interactions. A previous study conducted by Saccardi et al. [15] indicated that the substrate material did not significantly influence propolis’ work of adhesion. Biological adhesives often exhibit a minimal reliance on the chemical properties of the substrate [46]. It is our hypothesis that specific fluid components within propolis, such as mono- and sesquiterpenoids, may modify the interaction between the propolis sample and the substrate, resulting in adhesion that is nearly unaffected by the substrate’s characteristics.

### 4.2. Adhesion of Propolis on Bioinspired Surfaces

There are many recent papers on the anti-adhesive properties of various microstructures [47,48,49,50,51,52,53,54,55,56]. We wanted to investigate the influence of surface microstructures on propolis adhesion through adhesion experiments on various bioinspired substrates. Tests involving propolis on replicated mandibles and a smooth control substrate revealed that these structures did not reduce adhesion [15]. This possibly happened because, in the real mandible, the mandible surface is covered with a fluid, which was not replicated here. The role of the structures could be to evenly distribute this fluid.

Adhesion on the rough steel substrate was significantly lower compared to the smooth steel control. Irregular surface roughness likely reduced the actual contact area between the propolis sample and the substrate, as only the highest points made adhesive contact. In contrast, the Bénard cell structures on the lacquer surface did not significantly affect propolis adhesion when compared to the smooth control. These structures were larger and had a gentler slope than the previously mentioned structures, possibly allowing the propolis sample to adapt to the surface pattern, resulting in no reduction in contact area.

Hexagonal pins also did not reduce adhesion compared to the smooth Spurr control. Since these pins closely resembled the bee mandible model, it further supports the previous assumption that the structures on bee mandibles are not responsible for reduced propolis adhesion. In contrast, hexagonal dimples significantly reduced propolis adhesion. This could be due to the thin walls between the dimples hindering the formation of a tight contact between the propolis and the flat surface in sample valleys, resulting in a smaller contact area. The pins area fraction is 75% compared to only 25% of dimples [31]. Varenberg et al. [31] also found that adhesion and friction on dimples are significantly lower than on pins due to the reduced contact area. In comparison to the hexagonal dimples, the slight bulges found on the edges of some scales on the bee mandible were probably not high or sharp enough to prevent effective propolis contact with the scale surface.

It would be of interest to measure propolis adhesion on structured surfaces coated with a liquid layer that simulates the surface coating found on mandibular structures. Since propolis adhesion is significantly reduced even on smooth surfaces in wet conditions [11], we can anticipate an even more pronounced anti-adhesive effect on the microstructured surfaces covered with fluid. This expectation is based on the combination of surface structure and fluid, as previously demonstrated in water-filled silicone microstructures that prevent wetting by barnacle glue [57,58,59,60]. Furthermore, the spread of liquids on these structured substrates could be observed to confirm the assumption that the scales on bee mandibles aid in even distribution of the fluid coating the surface.

An effective anti-adhesive system based on a fluid layer evenly distributed by surface structures like those found on bee mandibles could have applications in various industries, for example in the woodworking industry, where resin-contaminated tools pose a significant problem, as cleaning is challenging and time-consuming. Similar systems involving slippery liquid-infused porous surfaces have been shown to be highly effective in reducing the adhesion of various substances such as ice [61] or biofilms [62].

## Figures and Tables

**Table 1:** Propolis adhesion on bioinspired surfaces. Work of adhesion and pull-off forces obtained from adhesion experiments (N = 5 propolis samples, n = 10 individual measurements per substrate). Mean values and standard deviations (s.d.) are given. Reference measurements on honeybee mandibles were reported by [15].

Substrate	N×n	Work of Adhesion [J/m^2^]		Pull-off Force [mN]	
		Mean	s.d.	Mean	s.d.
Honeybee mandible, unwashed	150	1.01	0.21	0.71	0.33
Steel, polished	50	2.29	0.82	1.98	0.51
Steel, rough	50	1.26	0.61	1.09	0.43
Lacquer, smooth (UV lacquer)	50	1.74	0.98	4.20	0.70
Lacquer, Bénard cells (UV lacquer)	50	1.99	1.07	3.76	0.60
Spurr, smooth	50	3.61	0.95	3.35	0.87
Hexagon dimples (Spurr)	50	1.99	0.64	1.99	0.55
Hexagon pins (Spurr)	50	3.28	1.38	2.31	1.08

## Data Availability

Data are contained within the article.
